# Is bilateral laminectomy and fusion superior to total laminectomy and fusion in two-level lumbar spinal stenosis?

**DOI:** 10.3389/fsurg.2026.1863550

**Published:** 2026-06-24

**Authors:** Xiaoyang Sun, Dichao Huang

**Affiliations:** 1 Department of Spine, Ningbo No.6 Hospital, Ningbo, Zhejiang, China; 2 Ningbo Clinical Research Center for Orthopedics, Sports Medicine & Rehabilitation, Ningbo, Zhejiang, China

**Keywords:** bilateral laminectomy and fusion, clinical effect, lumbar spinal stenosis, surgery, total laminectomy and fusion

## Abstract

**Background:**

This study aims to compare the clinical outcomes of bilateral laminectomy fusion (BLF) with total laminectomy and fusion (TLF) surgeries for patients with two-level lumbar spinal stenosis (LSS).

**Methods:**

A total of 124 eligible patients with lumbar spinal stenosis treated either with BLF or TLF between January 2023 and October 2025 at our hospital were enrolled in this study. Patients were divided into two groups according to their operation methods (BLF group and TLF group). All data were collected from their medical records. Data on clinically related factors, such as the Visual Analog Scale (VAS) score, Oswestry Disability Index (ODI), Japanese Orthopaedic Association (JOA) score for low back pain, and MacNab criteria, and operation-related factors such as hospital stay, operation time, blood loss, and day to walk, as well as complications, were collected and compared.

**Results:**

There were no significant differences in terms of age, gender, pain side and operation level, preoperative VAS score, NDI, and JOA score between the two groups of patients (*P* > 0.05). Patients in the BLF group showed a significantly reduced hospital stay, blood loss, and day to walk. Moreover, patients in the BLF group showed significantly better improvement in clinical factors such as the ODI, VAS score, and JOA score at each follow-up visit point than the TLF group (*P* < 0.05). The excellent/good rates (MacNab criteria) were 95% (57/60) in the BLF group and 91% (58/64) in the TLF group. With regard to complications, three patients suffered from nerve root irritation symptoms and two patients had mild spinal epidural tear in the TLF group, while nerve root irritation symptoms occurred in only one patient in the BLF group.

**Conclusions:**

BLF may provide better clinical outcomes than TLF in patients with LSS. Also, more studies are needed to further investigate the efficacy of BLF.

## Introduction

Lumbar spinal canal stenosis (LSS) is a common disease in elderly patients. The typical pathological changes in LSS denote a degenerative cascade initiated by intervertebral disc degeneration and height loss. This process alters spinal biomechanics and triggers compensatory, space-occupying changes (1), including hypertrophy and buckling of the ligamentum flavum, facet joint arthropathy with osteophyte formation, and concomitant lumbar disc bulging. As a result of these combined factors, the spinal canal and lateral recesses are narrowed (2), producing a characteristic “trefoil-shaped” canal and compression of the dural sac and nerve roots (3). This mechanical compression, particularly during lumbar extension, results in venous congestion and arterial insufficiency within the cauda equina. During walking, when metabolic demand increases, these changes lead to the characteristic symptoms of neurogenic claudication (4). These symptoms are relieved by lumbar flexion, which enlarges the spinal canal and reduces pressure on the neurovascular structures (5).

Surgical treatment is indicated for patients in whom conservative therapy has failed. The most commonly used surgical method for lumbar spinal stenosis is decompressive laminectomy with fusion (6). This procedure is widely regarded as the “gold standard” for LSS; however, it is associated with several disadvantages, including surgical morbidity and implant-related complications. Försth et al. reported outcomes in 123 patients with LSS treated with decompressive laminectomy and fusion, showing complications such as dural tears in 12 patients (11%), postoperative wound infections in 11 patients, and major adverse events (myocardial infarction, stroke, or thromboembolism) in three patients (7). Moreover, Regev et al. reported that 58.7% of patients experienced chronic back pain during a 5-year follow-up period after surgery (8). Chronic back pain is defined as persistent or recurrent pain following spinal surgery in the absence of identifiable causes such as infection, tumor, or implant failure, and with no indication for reoperation based on comprehensive clinical and imaging evaluation. A key diagnostic criterion is that the pain persists beyond the normal tissue healing period, typically for more than 3–6 months (9, 10).

To address these limitations, less invasive decompression techniques have been proposed, including bilateral laminectomy with fusion (BLF). Overdevest et al. reported that patients undergoing BLF had a lower incidence of iatrogenic instability and postoperative back pain (11).

However, to date, few studies have directly compared the clinical outcomes of unilateral laminectomy with fusion (BLF) and total laminectomy with fusion (TLF). It therefore remains unclear whether BLF can improve postoperative recovery and reduce complications compared with TLF. Accordingly, the aim of this study was to evaluate the therapeutic efficacy of these two surgical approaches. Specifically, we investigated whether BLF in patients with LSS could (1) provide better pain relief and reduce long-term back pain; (2) shorten the postoperative recovery period; and (3) improve clinical outcomes during follow-up.

## Methods

### Patients

This retrospective analysis was conducted using data from a prospectively maintained, single-center database, including patients who underwent TLF or BLF between January 2023 and October 2025 at Ningbo No.6 People's Hospital. Diagnosis was established based on clinical symptoms, physical examination, magnetic resonance imaging, and failure of at least 3 months of conservative treatment.

The inclusion criteria were as follows: (1) age ≥18 years; (2) diagnosis of two-level lumbar spinal stenosis; (3) failure of at least 3 months of conservative treatment; and (4) treatment with TLF or BLF with a minimum follow-up period of 6 months.

The exclusion criteria were: (1) prior lumbar spine surgery; (2) congenital or structural spinal abnormalities, including hemivertebra, block vertebra, or scoliosis; and (3) comorbid conditions that could affect clinical outcomes, such as tumors, rheumatoid arthritis, spinal infections, or severe cardiac disease.

This study was conducted in accordance with the Declaration of Helsinki and was approved by the Institutional Review Board and Ethics Committee of Ningbo No.6 Hospital. All patient data were anonymized prior to analysis. The requirement for written informed consent was waived because of the retrospective nature of the study.

### Surgery procedure

All patients were operated upon by the chief spine surgeon. The details of the surgical procedures are given in the following paragraphs.

### TLF group

After the patient was placed under general anesthesia and positioned prone on the surgical table, a standard posterior midline incision was made to expose the vertebral laminae and bilateral facet joints. Pedicle screws were inserted into the upper and lower vertebral bodies for subsequent stabilization. Total laminectomy, including removal of the spinous process, bilateral laminae, yellow ligament, and the medial portions of the facet joints, was meticulously performed to fully decompress the spinal canal and provide wide exposure of the dural sac and bilateral nerve roots. Following complete neural decompression, the nerve roots and dural sac were carefully retracted to access the disc space. The degenerative disc was thoroughly removed, and the cartilaginous endplates were prepared to create a vascularized bed for fusion. Then, on the right side, a cage filled with bone graft material was inserted into the disc space, typically placed to restore disc height and promote fusion. Finally, compression was applied via the pedicle screw–rod system (Medtronic, USA) to achieve rigid stabilization and promote interbody fusion. The surgical field was irrigated with normal saline, and a negative-pressure drain was placed deep to the fascia. The lumbodorsal fascia, subcutaneous tissue, and skin were subsequently closed in layers.

### BLF group

After successful general anesthesia, the patient was carefully positioned prone on positioning pads to keep the abdomen free, which can effectively reduce intra-abdominal pressure and minimize epidural venous bleeding. Following routine disinfection and draping, a standard posterior midline incision was created over the affected spinal segment. The skin, subcutaneous tissue, and lumbodorsal fascia were exposed sequentially. The paravertebral muscles were bluntly dissected using a periosteal elevator to expose the bilateral laminae, facet joints, and the base of the transverse process. Under precise guidance of a C-arm fluoroscope, six pedicle screws were inserted bilaterally into the vertebrae adjacent to the affected level.

**Figure 1:**
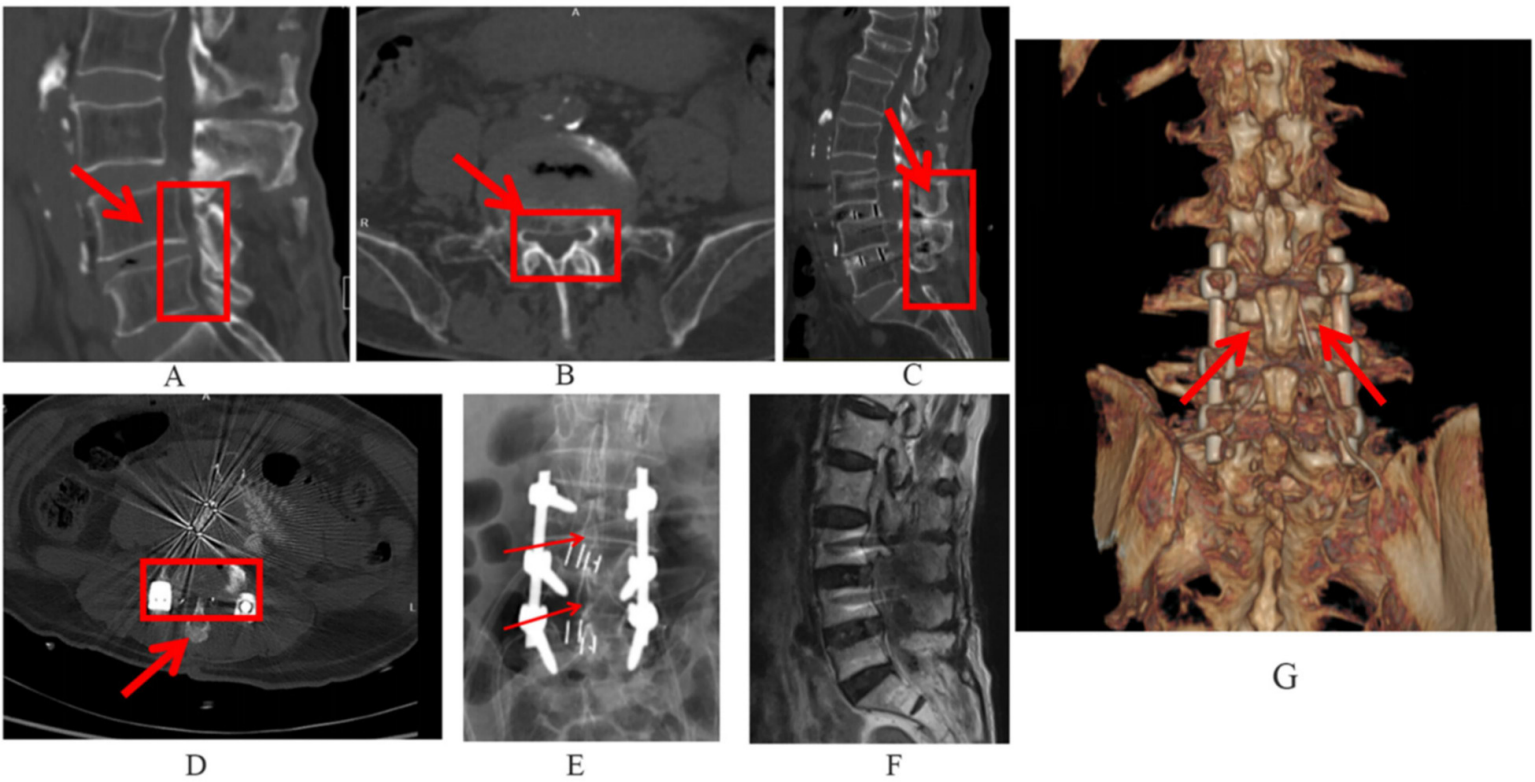
Preoperative and postoperative imaging of two-level lumbar spinal stenosis treated with bilateral laminectomy and fusion (BLF) from a representative 58-year-old male patient. **(B)** Axial preoperative computerized tomography (CT) demonstrating a trefoil-shaped canal and lateral recess stenosis. **(C)** Sagittal postoperative CT showing preservation of the spinous processes. **(D)** Axial postoperative CT demonstrating bilateral laminectomy. **(E, F)** Postoperative radiographs showing internal fixation and adequate decompression of the dural sac. **(G)** Three-dimensional CT reconstruction demonstrating the extent of bilateral laminectomy.

We completely resected the bilateral inferior articular process using a high-speed burr. If necessary, the medial portion of the superior articular process may also be partially resected. This maneuver widely opens the lateral recess and the posterior wall of the neural foramina, allowing for extensive exploration and thorough decompression of the central spinal canal and bilateral nerve roots, while also facilitating the release of any adherent nerve tissue. Normally, we insert a cage on the right side, and therefore, on the left side, only partial laminae, the inferior articular process, and the yellow ligament are removed. On the right side, after removing partial laminae, the inferior articular process, and the yellow ligament, the dural sac and bilateral nerve roots are meticulously identified and protected (12). They are gently retracted medially to expose the posterior annulus fibrosus. The degenerative nucleus pulposus is meticulously removed using nucleus pulposus forceps, curettes, and endplate shavers. The cartilaginous endplates are thoroughly curetted down to the bleeding bony endplate to create a well-vascularized bed conduction for fusion (13). Autologous bone was harvested from the decompression site (including the resected facets and laminae) and morselized into small chips and mixed with an appropriate amount of artificial bone graft. These bone grafts were tightly impacted into the anterior and middle third of the disc space. Then, one suitably sized interbody cage filled with cancellous bone was obliquely inserted to restore disc height and maintain neural foramen volume. Finally, moderate compression was applied across the instrumented segment by two rods, which can enhance the initial stability of the cage before all locking nuts are tightened. The wound was irrigated with normal saline, a negative-pressure drain was placed deep inside the fascia, and the lumbodorsal fascia, subcutaneous tissue, and skin were closed layer by layer (Figure 1).

The postoperative rehabilitation protocol was similar in both groups of patients, and a lumbar brace was used by them for 12 weeks.

### Outcome evaluation

Baseline characteristics such as age, body mass index (BMI), gender, smoking status, and diabetes were collected from the patients’ medical records, and data on operation-related parameters (hospital stay, operation time, blood loss, and day to start walking), clinical outcomes [Visual Analog Scale (VAS) score, Oswestry Disability Index (ODI), and Japanese Orthopaedic Association (JOA) score], as well as complications, were also collected.

The ODI was employed to assess the level of functional disability as perceived and reported by the patients themselves (14). In addition, the VAS was used for patients to rate the intensity of their current pain, encompassing both lower back discomfort and radiating leg pain (radicular pain) (15). The JOA score was typically recorded by the physician, based on objective clinical findings obtained during the physical examination, with the aim of quantifying the overall severity of the disease (16). The levels of C-reactive protein (CRP) were also compared in the two patient groups, because this indicator can effectively reflect the condition of tissue damage (17).

Postoperative complications were documented based on a review of patients’ medical records and subsequently analyzed. These complications included conditions that either delayed recovery or required medical intervention, such as incision suppuration, cerebrospinal fluid leakage, fixation failure, chronic back pain, and nerve root irritation symptoms. In addition, local inflammatory signs at the incision site—such as redness, swelling, and pain—were recorded.

### Statistical analysis

Statistical Package for Social Sciences 25.0 (IBM Corp., Armonk, NY, USA) was used for the statistical analysis. Data were expressed as mean ± SD. The Shapiro–Wilk test was used to determine the normality of continuous data. For comparisons between the two groups (BLF vs. TLF), an independent sample *t*-test was used for normally distributed data and the Mann–Whitney *U* test for non-normally distributed data. The categorical data were compared using the chi-square test. A value of *P* < 0.05 was considered statistically significant.

## Results

### Baseline characteristics

A total of 124 patients diagnosed with two-level lumbar spinal stenosis met the inclusion criteria for this study. Their medical records were retrospectively reviewed, and the relevant data were compared between the two groups, as summarized in Table 1. No statistically significant differences were observed between the groups in terms of age, gender distribution, BMI, smoking status, or prevalence of diabetes (*P* > 0.05 for all). However, several clinical parameters showed significant differences. Specifically, the BLF group had a significantly shorter hospital stay compared with the TLF group (7.1 ± 1.4 days vs. 8.5 ± 1.1 days; *P* < 0.05). In addition, the time to first ambulation postsurgery was significantly longer in the TLF group (2.4 ± 0.5 days) than in the BLF group (2.1 ± 0.8 days; *P* < 0.05). On the other hand, intraoperative blood loss was significantly greater in the TLF group (410.5 ± 77.5 mL) than in the BLF group (345.3 ± 105.3 mL; *P* < 0.05). Furthermore, the operative time was also significantly longer in the BLF group (134.7 ± 19.8 min) than in the TLF group (120.1 ± 21.3 min; *P* < 0.05).

**Table 1:** The baseline characteristics of patients from the two groups.

	BLF group	TLF group	*P*-value
Number	60	64	
Gender			0.51
Male	29	30	
Female	31	34	
Age (year)	62.4 ± 8.1	63.3 ± 7.5	0.55
BMI (kg/m^2^)	22.8 ± 0.5	22.5 ± 0.7	0.003
smoke(yes/no)	33/27	34/30	0.49
Diabetes (yes/no)	21/39	24/40	0.46
Hospital stay (day)	7.1 ± 1.4	8.5 ± 1.1	<0.05
Operation time (min)	134.7 ± 19.8	120.1 ± 21.3	<0.05
Blood loss (mL)	410.5 ± 77.5	345.3 ± 105.3	<0.05
Day to walk (day)	2.1 ± 0.8	2.4 ± 0.5	0.001

### Clinical outcomes

**Figure 2:**
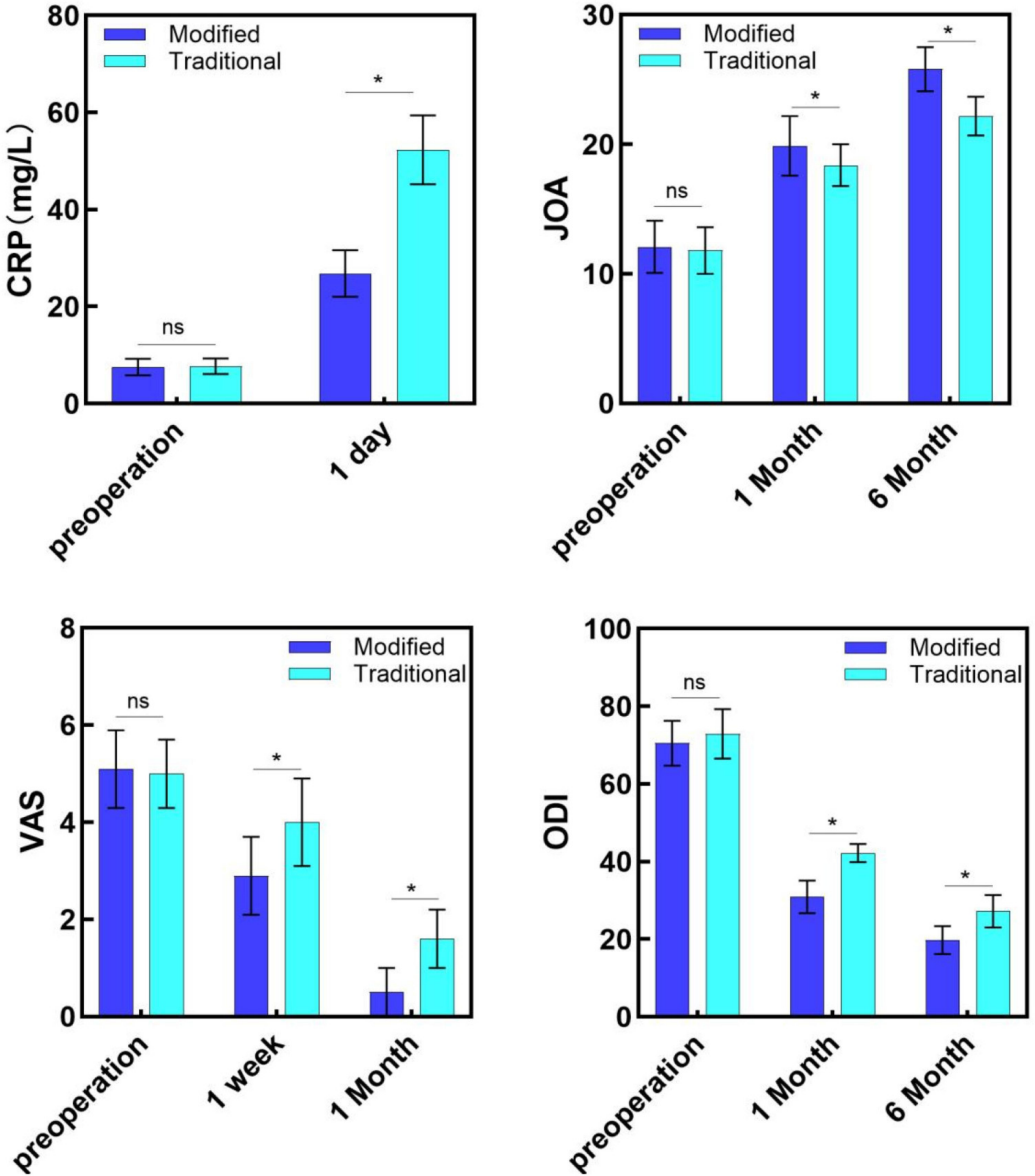
Comparison of inflammatory markers and clinical outcomes between traditional total laminectomy with fusion (TLF) and modified bilateral laminectomy with fusion (BLF). *P < 0.05.

The clinical outcomes are provided in Table 2. There were no significant differences in preoperative JOA score, VAS score, and ODI between the two groups (all *P* > 0.05). Whereas, we found that the BLF group showed significantly better improvement in the VAS score (1 week:2.9 ± 0.8 vs. 4.0 ± 0.9; 1 month:0.5 ± 0.5 vs. 1.6 ± 0.6), JOA score (1 month:19.9 ± 2.3 vs. 18.4 ± 1.6; 6 months: 25.8 ± 1.7 vs. 22.2 ± 1.5), and ODI (1 month:30.9 ± 4.2 vs. 42.2 ± 2.3; 6 months:19.8 ± 3.6 vs. 27.2 ± 4.2) compared with the TLF group (All *P* < 0.05). In addition, the CRP levels were significantly lower in the BLF group than in the TLF group immediately after the operation (26.8 ± 4.8 mg/L vs. 52.3 ± 7.1 mg/L; *P* < 0.05). To improve the clarity and visual interpretation of the data, the clinical outcomes are presented graphically (Figure 2).

**Table 2:** The clinical outcomes of patients from the two groups.

	BLF group	TLF group	*P*-value
Number	60	64	
VAS			
Preoperation	5.1 ± 0.8	5.0 ± 0.7	0.72
1-week follow-up	2.9 ± 0.8	4.0 ± 0.9	<0.05
1-month follow-up	0.5 ± 0.5	1.6 ± 0.6	<0.05
ODI			
Preoperation	70.5 ± 5.8	72.9 ± 6.4	0.03
1-month follow-up	30.9 ± 4.2	42.2 ± 2.3	<0.05
6-month follow-up	19.8 ± 3.6	27.2 ± 4.2	<0.05
JOA			
Preoperation	12.1 ± 2.0	11.8 ± 1.8	0.34
1-month follow-up	19.9 ± 2.3	18.4 ± 1.6	<0.05
6-month follow-up	25.8 ± 1.7	22.2 ± 1.5	<0.05
CRP			
Preoperation	7.5 ± 1.7	7.7 ± 1.6	0.65
Postoperation	26.8 ± 4.8	52.3 ± 7.1	<0.05

With regard to complications and the MacNab criteria presented in Table 3, the BLF group demonstrated superior outcomes, with 95% of patients achieving excellent or good results according to the MacNab criteria, compared with 90.6% in the TLF group (*P* < 0.05). These findings further support the clinical advantages of BLF in reducing postoperative morbidity and enhancing functional recovery. Moreover, the BLF group had a significantly lower overall complication rate (3.3% vs. 18.8%, *P* < 0.05). In the BLF group, postoperative complications were minimal, with only one case of nerve root irritation and one epidural tear, both of which were successfully managed with conservative treatment. In contrast, the TLF group exhibited a higher incidence and greater variety of complications, including incision infection (two patients), fixation loosening (one patient), chronic back pain (four patients), nerve root irritation (three patients), and epidural tear (two patients). These findings suggest that BLF is associated with a lower complication rate and reduced postoperative morbidity compared with TLF.

**Table 3:** The MacNab criteria and complications in the two groups of patients.

	BLF group	TLF group	*P*-value
MacNab			<0.05
Excellent	48	41	
Good	9	17	
Fair	3	5	
Poor	0	1	
Complication			<0.05
Incision infection	0	2	
Fixation loosen	0	1	
Chronic back pain	0	4	
Nerve root irritation	1	3	
Epidural tear	1	2	

## Discussion

LSS arises from a multifactorial pathomechanism characterized by progressive narrowing of the central spinal canal, lateral recess, and intervertebral foramina (18). This reduction in available space results from both congenital developmental anomalies and acquired degenerative changes of the lumbar spine, including intervertebral disc degeneration, protrusion, and height loss, hypertrophy and arthrosis of the facet joints, thickening, fibrosis, and calcification of the ligamentum flavum, as well as osteophyte formation and spinal instability (19). These structural alterations induce both static and dynamic mechanical compressions of the dural sac, cauda equina, and nerve roots, impairing neural microvascular perfusion and triggering ischemia, edema, and inflammatory responses within neural tissues. Consequently, the resultant neurogenic claudication, radicular pain, and neurological deficits constitute the clinical syndrome of lumbar spinal stenosis.

Multiple risk factors contribute to the development of acquired degenerative changes in the lumbar spine that ultimately lead to spinal stenosis (20). (1). Advanced age is the most significant factor, characterized by progressive cellular senescence, disc dehydration, and loss of structural integrity, which promote disc degeneration, facet joint hypertrophy, ligamentum flavum thickening, and osteophyte formation. (2). Mechanical overload, including repetitive lumbar loading, heavy physical labor, and prolonged static postures, sarcopenia, obesity, or an elevated body mass index, increases axial loading and accelerates degenerative processes (21). (3). Non-modifiable risk factors include a history of spinal injury or surgery that leads to secondary instability and adaptive hypertrophy, a genetic predisposition that affects extracellular matrix metabolism and cartilage integrity, and lifestyle factors such as smoking that impairs vertebral endplate perfusion and disc nutrition (22). Collectively, these factors accelerate degenerative remodeling of the lumbar spine, reduce the dimensions of the spinal canal and lateral recess, and increase the likelihood of the development of lumbar spinal stenosis (23).

Currently, TLF remains the standard surgical treatment for LSS. Although numerous studies have demonstrated the effectiveness of TLF, the procedure is associated with several complications, including adjacent segment disease (24) and chronic postoperative back pain (25). To address these limitations, the concept of decompression with preservation of the spinous process has been proposed (26).

In this study, we retrospectively compared clinical outcomes between two groups of patients, namely, the BLF group and TLF group. There were no significant differences between the groups in baseline characteristics, including age, sex, BMI, smoking status, and prevalence of diabetes (all *P* > 0.05). However, we found out that the BLF group showed significantly better improvement in the JOA score, ODI, and VAS score during a follow-up visit. Both hospital stay (7.1 ± 1.4 days in the BLF group vs. 8.5 ± 1.1 days in the TLF group; *P* < 0.05) and the time to first ambulation postsurgery (2.1 ± 0.8 days in the BLF group vs. 2.4 ± 0.5 days in the TLF group; *P* < 0.05) were significantly shorter in the BLF group than in the TLF group. In addition, intraoperative blood loss was significantly greater in the TLF group (410.5 ± 77.5 mL) than in the BLF group (345.3 ± 105.3 mL; *P* < 0.05). Furthermore, the operative time was significantly longer in the BLF group (134.7 ± 19.8 min) than in the TLF group (120.1 ± 21.3 min; *P* < 0.05). Similar results were also reported by Li et al. in their study. They compared unilateral laminotomy with conventional laminectomy and found that the unilateral laminotomy group of patients had significantly lower VAS scores for back pain and ODI scores than the conventional laminectomy group of patients (27). Moreover, in this study, we compared the CRP levels between the two groups, because the CRP level was considered an indicator of tissue damage (28). We found that the CRP levels were significantly lower in the BLF group than in the TLF group immediately after operation (26.8 ± 4.8 mg/L vs. 52.3 ± 7.1 mg/L;*P* < 0.05). This indicator indicates that BLF causes less damage to human tissues than TLF and is beneficial for the patient in their road to recovery. With regard to complications and MacNab criteria, there were significantly more patients in the TLF group who suffered from complications than in the BLF group. Also, the excellent and good rates were 95% in the BLF group and 90.6% in the TLF group.

## Conclusions

This study indicates that BLF may provide better clinical outcomes than TLF in patients with two-level lumbar spinal stenosis, including shorter hospital stay, earlier ambulation, and reduced surgical trauma, although it may require a longer operative time. This study contributes to the limited comparative evidence on BLF vs. TLF, suggesting that BLF represents a less invasive yet clinically effective alternative. These findings provide practical guidance for surgical decision-making and support the preferential use of BLF in appropriately selected patients, although further prospective and multicenter studies are needed to validate its long-term benefits.

## Limitations

This study has two major limitations. First, there is a potential risk of selection bias due to its retrospective, single-center design and relatively small sample size. Second, subgroup analyses based on age were not performed; advanced age may influence clinical outcomes.

## Data Availability

The original contributions presented in the study are included in the article/Supplementary Material, and further inquiries can be directed to the corresponding author.
